# Shaping communicative colour signals over evolutionary time

**DOI:** 10.1098/rsos.160728

**Published:** 2016-11-30

**Authors:** Alison G. Ossip-Drahos, José R. Oyola Morales, Cuauhcihuatl Vital-García, J. Jaime Zúñiga-Vega, Diana K. Hews, Emília P. Martins

**Affiliations:** 1 Department of Biology, Indiana University, Bloomington, IN, USA; 2 Center for the Integrative Study of Animal Behavior, Indiana University, Bloomington, IN, USA; 3 Department of Biology, University of North Georgia, Oakwood, GA 30566, USA; 4 Departamento de Ciencias Veterinarias, Instituto de Ciencias Biomédicas, Universidad Autónoma de Ciudad Juárez, Ciudad Juárez, Mexico; 5 Departamento de Ecología y Recursos Naturales, Facultad de Ciencias, Universidad Nacional Autónoma de México, Mexico City, Mexico; 6 Department of Biology, Indiana State University, Terre Haute, IN, USA

**Keywords:** sex differences, animal coloration, visual communication, natural selection, sexual selection, *Sceloporus* lizards

## Abstract

Many evolutionary forces can shape the evolution of communicative signals, and the long-term impact of each force may depend on relative timing and magnitude. We use a phylogenetic analysis to infer the history of blue belly patches of *Sceloporus* lizards, and a detailed spectrophotometric analysis of four species to explore the specific forces shaping evolutionary change. We find that the ancestor of *Sceloporus* had blue patches. We then focus on four species; the first evolutionary shift (captured by comparison of *S. merriami* and *S. siniferus*) represents an ancient loss of the belly patch by *S. siniferus*, and the second evolutionary shift, bounded by *S. undulatus* and *S. virgatus*, represents a more recent loss of blue belly patch by *S. virgatus*. Conspicuousness measurements suggest that the species with the recent loss (*S. virgatus*) is the least conspicuous. Results for two other species (*S. siniferus* and *S. merriami*) suggest that over longer periods of evolutionary time, new signal colours have arisen which minimize absolute contrast with the habitat while maximizing conspicuousness to a lizard receiver. Specifically, males of the species representing an ancient loss of blue patch (*S. siniferus*) are more conspicuous than are females in the UV, whereas *S. merriami* males have evolved a green element that makes their belly patches highly sexually dimorphic but no more conspicuous than the white bellies of *S. merriami* females. Thus, our results suggest that natural selection may act more immediately to reduce conspicuousness, whereas sexual selection may have a more complex impact on communicative signals through the introduction of new colours.

## 1. Introduction

Communicative signals are shaped by a complex combination of evolutionary forces that can shift over time, and we can use comparative studies to explore how and when signals respond to different evolutionary forces [1–4]. In some cases, natural and sexual selection act on different elements of the signal. For example, dorsal colours are often shaped by natural selection to make an animal cryptic to predators, whereas the impact of sexual selection is focused on signalling colours that are dramatically displayed only to conspecifics [5,6]. In other cases, natural and sexual selection act differentially on senders and receivers or at different points in time. For example, the sensory bias hypothesis predicts that natural selection first impacts receiver sensory systems, and is then followed by sexual selection acting on signal production (reviewed in [7]). Here, we use phylogenetic analyses and reflectance spectrometry measurements of four representative *Sceloporus* lizard species to explore changes in a communicative signal over long periods of evolutionary time.

Comparative studies of colour reveal that sexual selection is often the primary driver of sexual dimorphism in birds [2,3] and lizards [1,4], whereas the response to natural selection is often manifested through background matching [5,8]. By gaining or losing colours, animals can become more or less conspicuous to visually oriented predators [6,9], and comparative studies indicate tight evolutionary links between habitat and evolutionary shifts in coloration [10,11]. However, natural and sexual selection can act simultaneously or alternately over long periods of time when shaping sexual dimorphism. For example, in grackles [12] and fairy wrens [13], sexual selection appears to act continuously on males, resulting in steady divergence in colour over time, whereas the response to natural selection appears in bursts, facilitating frequent increases and decreases in the rates of change in female colour. In strawberry poison frogs, both natural and sexual selection shape dorsal coloration [14], but sexual selection predominantly shapes brightness over short periods of time (within populations), whereas natural selection has a larger impact on brightness over longer periods of time (across populations, [15]).

Phylogeny can predict behavioural evolution effectively [16–18] and can be a powerful, under-used, tool for understanding the evolutionary timing of behavioural events [19,20]. Reconstructing ancestral patterns of trait evolution allows us to determine the evolutionary lability of traits and the relative timing of events. For example, the sensory bias hypothesis has been tested by reconstructing the macroevolutionary patterns of traits and perception of traits to infer the sequence of historical events (reviewed in [21]). Reconstructing ancestral phenotypes can also allow us to infer the relative timing of evolutionary events and to estimate the impact of phylogenetic inertia in shaping morphological and behavioural variation. For example, ancestral reconstructions of colours in grackles reveal higher rates of evolutionary change in females when compared with males [12].

Phylogenetic considerations of animal colours are difficult, in part, because human vision may underestimate the spectral variation in animal colour and misrepresent the conspicuousness of a colour from the perspective of another animal. Endler's [22] early insights into the importance of using objective measures of colour are now routinely coupled with Vorobyev & Osorio's [23] visual model, which effectively estimates the contrast between an animal and its habitat while taking into account the sensory system of the receiver (see also [24]). Incorporating objective measures of the habitat has led to several important findings. For example, Cole & Endler [25] showed that the colour preferences of female guppies depend on the colours of the water through which they view males. Similarly, perceived sexual dichromatism of Eurasian perch increases with water transparency [26], and *Anolis* lizards in different light environments have evolved colour signals that maximize detection [27,28]. Incorporating receiver sensory systems is also clearly important, for example, leading to findings that male birds with highly attractive colour ornaments provide less paternal care then do less attractive males [29,30], and that contrary to early beliefs, the evolution of dichromatism in songbirds is shaped by changes in female, rather than male, plumage [12]. Viewing angles and polarization can also play important roles, as in butterflies that alter their iridescent signals with every wing beat [31] and in ocean fish that remain cryptic to predators while visible to conspecifics because of the direction from which they are viewed [32].

Here, we use a comparative analysis of *Sceloporus* belly colours to ask about the relative timing of evolutionary change in colour. Lizards in the genus *Sceloporus* present an excellent opportunity to explore patterns of evolutionary shifts in colour, as there are repeated episodes in which males have lost the blue belly patches typical of the genus [33], and which are used during the agonistic male–male encounters [33–35] that drive sexual selection. Expression of the blue belly patches typical of *Sceloporus* males is mediated by the action of sex hormones during development [36], and is associated with a dense layer of abdominal melanin that underlies the reflecting iridophore layer [37]. Blue can be lost, and other colour elements added to the belly patch with relatively small changes in the production of androgen or melanocortin hormones or their receptors [38,39]. By comparing the belly colours of different species of *Sceloporus*, we can begin to understand how changes in colour occur over long periods of evolutionary time.

Using a recent phylogeny [40], we begin by reconstructing ancestral patterns of ventral coloration for the genus as a whole. We then focus on two evolutionary losses of the belly patches, conducting a detailed spectral analysis of the belly colours of four species. Differences between one pair of species reflect a loss of blue that occurred a very long time ago near the root of the phylogeny, whereas the second pair of species represents a loss of blue that occurred much more recently in evolutionary time. We conduct a spectral analysis of belly colours in these four species, relating the colours to their backgrounds and to the sensory systems of potential receivers. By comparing conspicuousness and sex differences of these four species, we can develop hypotheses about the timing and impact of different evolutionary forces over long periods of time.

## 2. Methods

### 2.1. Ancestral state reconstruction

To gain insights into the timing of large evolutionary shifts in colour (i.e. recent versus ancient shifts), we reconstructed the ancestral states for male belly colour along a recent, time-calibrated phylogeny for the family Phrynosomatidae [40]. Previous ancestral reconstructions indicate that the common ancestor of *Sceloporus* lizards likely had blue patches, and the observed white bellies in some species represent a loss of blue coloration [33]. However, more recent phylogenies place a clade of white-bellied species close to the base of the phylogeny [40–42], thus potentially changing the ancestral reconstruction at the root of the tree. Here, we pruned Wiens *et al*.'s [40] phylogeny to include only species in the genus *Sceloporus* and *Urosaurus* (i.e. the genus' closest outgroup; four *Urosaurus* species, 77 *Sceloporus* species).

Following Ossip-Klein *et al*. [11], we then identified presence/absence of blue belly patches as compiled by Martins [43] and Wiens [33], for a total of 11 *Sceloporus* species that have lost the patch: *S. bicanthalis, S. carinatus, S. chaneyi, S. cozumelae, S. edwardtaylori, S. horridus albiventris, S. megalepidurus, S. siniferus, S. squamosus, S. utiformis* and *S. virgatus*. We used stochastic character mapping with an underlying Bayesian assumption to estimate ancestral states and to infer the relative timing of the evolutionary shifts on the branches of the phylogeny [44–46]. To incorporate uncertainty about model choice, we fit both a single rate model across the phylogeny and a two-rate model [47], calculating the average rates over 1000 mappings, using the Akaike weights from the mean AICc scores. All of the statistical analyses were conducted in R [48], using the ‘phytools’ [49] and ‘geiger’ packages [50].

### 2.2. Choice of species for colour measurements

We then focused on two evolutionary shifts in which the blue belly patches have been lost, represented by four species. The first evolutionary shift (captured by comparison of *S. merriami* and *S. siniferus*) is more basal in the phylogeny, representing an ancient loss of the belly patch in the ancestor of the clade represented by *S. siniferus*. The second evolutionary shift, bounded by *S. undulatus* and *S. virgatus*, occurred more recently in evolutionary time, with *S. virgatus* representing a recent loss of the blue belly patch. These four species also represent a diversity of habitats in which *Sceloporus* are found. We measured *S. merriami* (*n* = 31 females, 24 males) on the steep, reddish-grey, rock walls of a slot canyon in the Chihuahua desert in the Big Bend Ranch State Park, TX, USA and found *S. siniferus* (*n* = 27 females, 28 males) in the thick green and brown vegetation of a semi-deciduous tropical rainforest at the Huatulco National Park, Mexico. We measured *S. undulatus* (*n* = 20 females, 20 males) on brown tree trunks and grey rocks on the shores of Lake Monroe, Indiana, USA and *S. virgatus* (*n* = 29 females, 36 males) on rocks along dry creek beds in the oak-pine woodlands of the Chiricahua Mountains in southeastern Arizona, USA. All lizards were measured between May and August 2012, during their peak active periods.

### 2.3. Colour measurements

To the human eye, *S. merriami* and *S. undulatus* males have colourful belly patches, whereas females have plain white bellies. In contrast, in *S. siniferus* and *S. virgatus*, both males and females have white bellies. Here, we used a spectrometer to measure these differences objectively. In doing so, we also take into account reflectance in the ultraviolet and fine-scale differences in colour to which diurnal lizards may be highly sensitive [39,51]. Specifically, we measured the spectral reflectance of lizard bellies relative to a WS-1-SL white reflectance standard with Spectralon (Ocean Optics Inc., Dunedin, FL), using a USB2000 + miniature fibre optic spectrometer (Ocean Optics Inc., Dunedin, FL) connected to a xenon lamp (Ocean Optics PX-2), a reflectance probe (Ocean Optics R200-7) and a laptop computer running Ocean Optics SpectraSuite software. The end of the probe was fitted with a light-proof black tip cut at a 45° angle (StellarNet, Tampa, FL) to maintain a consistent angle between the probe and the lizard. We took measurements across the spectrum of 300–700 nm wavelengths (*λ*), as this broad range includes wavelengths known to be visible to diurnal lizards (reviewed in [39]). We recalibrated the spectrometer after every third measurement.

We took three spectral reflectance measurements from the centre of the left ventral signalling patch (or white belly) of each lizard, and used the mean of the three measurements for all analyses. *Sceloporus merriami* have both blue and green elements to their belly patches, so we took separate measurements from the widest part of the left blue patch and the widest part of the left green patch. All lizards were measured within a few hours of capture, marked to prevent repeated sampling, and then released at the site of capture. From the side (the perspective of a conspecific or predator lizard), the substrate is the background against which a lizard belly patch is viewed. Thus, to characterize habitat colour, we took three measures of a representative sample of the primary substrate upon which we found most of the lizards (rock or log) and averaged to obtain a single measure. Although we found remarkably little variation in the colour of the substrates, we chose a representative sample of substrate measures based on where most of the lizards were found during their peak hours of activity.

### 2.4. Statistical analyses

We estimated conspicuousness from the perspective of a diurnal lizard by running the lizard spectral measures through a version of the Vorobyev & Osorio [23] visual model (see also [52–54]) of a tetrachromatic, diurnal lizard (*Crotaphytus dickersonae*; [55]; J. Macedonia 2015, personal communication). Briefly, for each photoreceptor type, this model calculates the relative quantum catch, taking into account the light entering the eye and the spectral sensitivities of the photoreceptors (including lens, ocular media, oil droplet absorbance and visual pigment absorbance). For tetrachromatic animals, this model projects the colour of objects as viewed under ambient light into a tetrahedral colour space, where distances in colour space are proportional to conspicuousness. A just notable difference (JND) greater than 1 indicates that colours can be discriminated from the background, with higher values being more easily distinguished (see [52–54] for details). We followed Martin *et al*. [56] in assuming that receptor noise does not depend on light intensity, using a Weber fraction of 0.05 as used for amphibians [53] and a measure of standard daylight irradiance (D65 spectrum, [57]). Following Macedonia *et al*. [55], we assumed all cone classes contributed equally to perception. Because published information on the retinal cone sensitivities of *Sceloporus* lizards is not available, we use normalized absorbances of the visual pigments and oil droplets of another lizard species, *Crotaphytus dickersonae*, to estimate conspicuousness from the perspective of a potential predator ([55]; J. Macedonia 2015, personal communication). *Sceloporus* are eaten by a wide variety of aerial and terrestrial predators, including larger lizards such as the *Crotaphytus* used in our visual model. Because lizard visual pigments tend to be conserved [58], these pigments and oil droplets are likely to be also similar to those of *Sceloporus*. Thus, we do not distinguish between conspicuousness from the perspective of predator as opposed to conspecific. All visual model calculations were run using Avicol v. 6 software [59].

Visual model calculations only yield a single value for conspicuousness without taking into account different parts of the spectral curve. Thus, to describe colour measures in more detail, we also considered the raw spectral curves. We divided the area under each spectral curve into 20 segments, each representing reflectance for 20 nm of the spectrum. We then used principal components analysis (PCA) to reduce those 20 measures (for both sexes and all four species simultaneously) into composite variables (as in [60–62]).

We compared conspicuousness of males of the species associated with a recent loss of the colourful belly patch (*S. virgatus*) with the species associated with a more ancient loss (*S. siniferus*) both directly and in the context of their colourful-bellied congeners. We used a combination of ANOVA (with JND scores) and MANOVA (with PC axes) to estimate the relative impacts of clade (ancient or recent loss of colour), colour (plain white or colourful) and their interaction, followed by Tukey *post hoc* tests for specific comparison of the two white-bellied species. We further compared the conspicuousness of male bellies to that of female bellies, adding a third factor for sex and a three-way interaction effect to the ANOVA and MANOVA models. Although other interpretations are possible, we chose these as starting points for generating hypotheses.

For each model, we examined residuals to confirm that the model conformed to the usual homoscedasticity and normality assumptions. All of the statistical analyses were conducted in R, using the basic and ‘psych’ packages [48].

## 3. Results

### 3.1. Repeated evolutionary shifts from blue to white bellies

**Figure 1.**
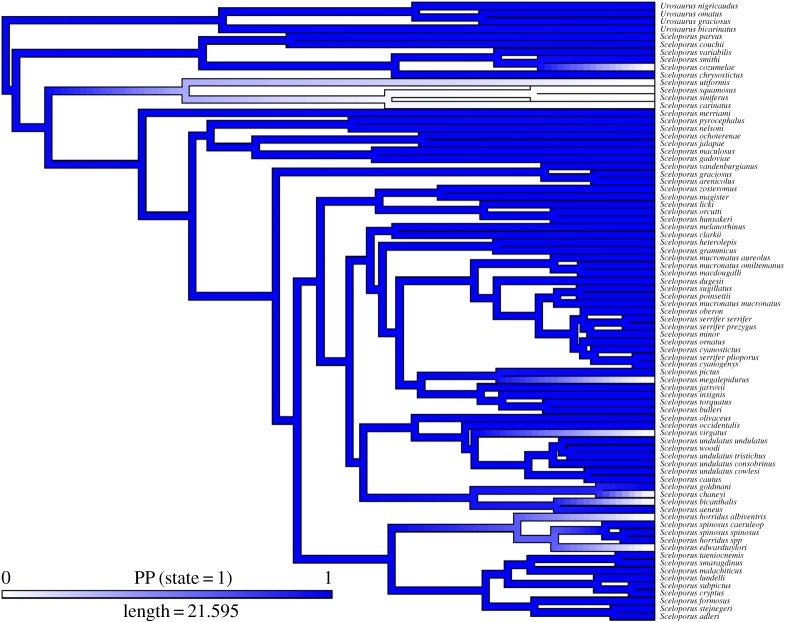
Time-calibrated phylogeny showing an average of 1000 mappings of ancestral states of belly colour for 77 species of *Sceloporus* (and four species of *Urosaurus*) using stochastic character mapping. Blue indicates presence of blue patches, whereas white indicates absence of blue patches. Length is in units of millions of years.

Blue belly patches probably first arose near or just before the root ancestor of *Sceloporus* (figure 1). The genus' closest relatives (*Urosaurus* and *Petrosaurus*) have blue belly patches, whereas a slightly more distant relative (*Uta*) has white bellies and coloured throat patches, suggesting that the blue belly patches initially arose before the split of *Urosaurus*, *Petrosaurus* and *Sceloporus*. There is support for the simpler, one-rate model, based on Akaike weights. Akaike weights were similar but slightly lower for the one-rate model (AICc = 61.69) compared with the two-rate model (AICc = 62.60), indicating that the one-rate model is sufficient. We thus report results from the one-rate evolutionary model. The root ancestor of *Sceloporus* very likely had blue patches (support for blue basal node = 98%). There were seven evolutionary losses of blue belly patches (based on posterior probabilities greater than 75%), and no reversals. Stochastic character mapping confirmed that the loss of the blue belly patches in *S. siniferus* occurred much longer ago than did the loss of blue colour in *S. virgatus* (figure 1).

### 3.2. White bellies from recent shift are cryptic; those from ancient shift are conspicuous

**Figure 2.**
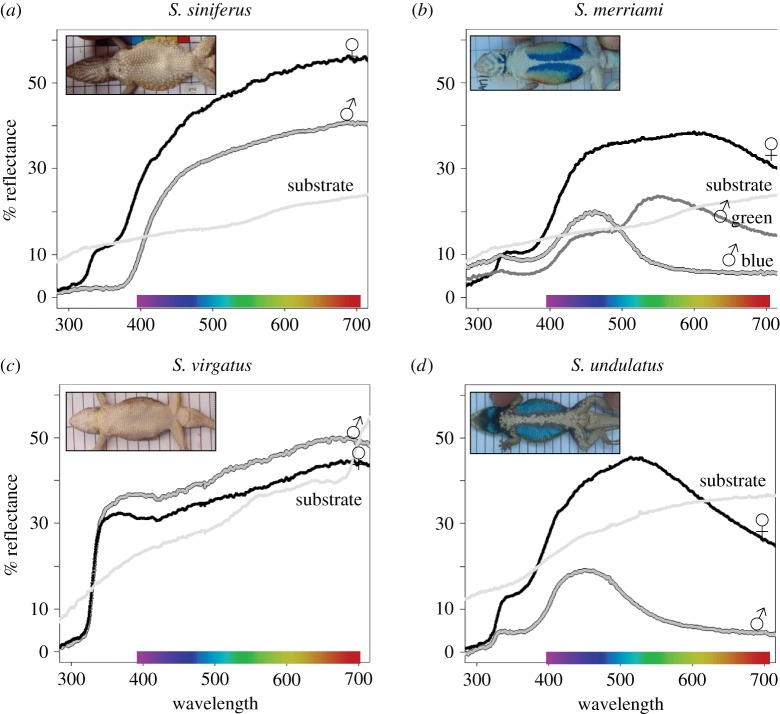
Representative spectral reflectance curves for males (♂, grey line), females (♀, black line), and a representative substrate (rocks or logs, light grey line) for each species. There are two grey lines for *S. merriami* males because these males have both blue and green components in their belly patches. The two top panels (*a,b*) are for the two species bounding an older evolutionary loss of the blue belly patch, and the bottom panels (*c,d*) are for the two species bounding a more recent loss. Photographs are of the ventral surfaces of a single representative male for each species.

**Figure 3.**
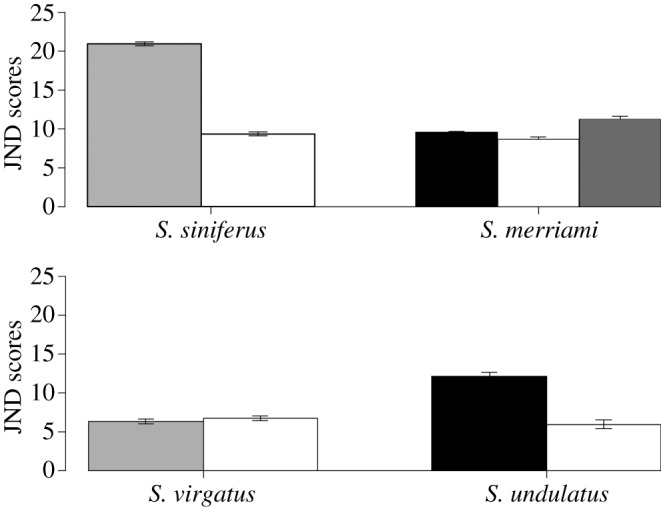
Average just notable differences (JNDs) from Vorobyev and Osorio visual model [23]. Higher JNDs mean that lizards are more conspicuous to conspecifics. For the white-bellied species, *S. siniferus* and *S. virgatus*, grey bars represent JND scores for males, while white bars represent JND scores for females. For the species with blue-bellied males, black bars are for males and white bars represent females. The dark grey bar for *S. merriami* indicates the JND scores for the green part of the male belly patches. Error bars indicate one standard error.

Although the bellies of both *S. siniferus* and *S. virgatus* males appear white to the human eye, the bellies of *S. siniferus* males were much more conspicuous than those of *S. virgatus* males when measured objectively (figure 2). When filtered through a lizard visual model, male *S. virgatus* bellies (recent loss of blue) were relatively inconspicuous, and much less conspicuous (i.e. more similar to their substrates) than were male *S. siniferus* bellies (ancient loss of blue; figure 3). *Sceloporus siniferus* males were actually the most conspicuous of all four species, whereas *S. virgatus* males were the least conspicuous. This led to a significant interaction between clade and colour in terms of conspicuousness as perceived by a lizard receiver (JNDs: *F*_1,95_ = 81.9; *p* < 0.001), that was further supported by a significant clade effect (*F*_1,95_ = 139.8; *p* < 0.001); the first clade (*S. merriami* and *S. siniferus*) that represents an ancient loss of the belly patch, was more conspicuous than the second clade (*S. undulatus* and *S. virgatus*) that represents a more recent loss of blue belly patch by *S. virgatus.* The colour main effect was not significant (*F*_1,95_ = 0.4; *p* = 0.52), because male conspicuousness of *S. merriami* blue but not green elements to the patches was intermediate to that of the two white-bellied species.

**Figure 4.**
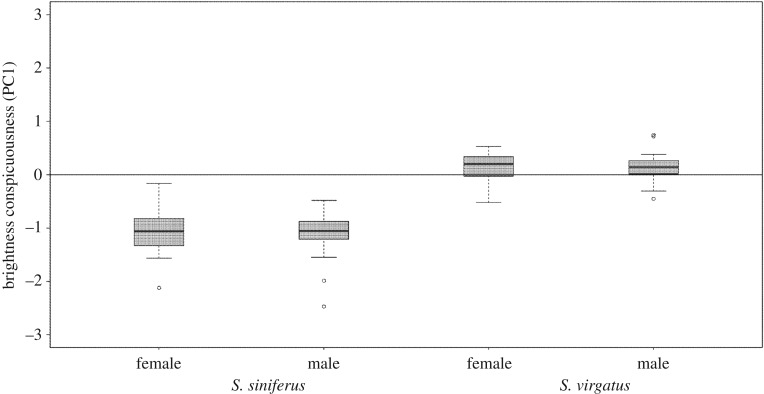
*S. siniferus* male bellies are more conspicuous (i.e. more different from the substrate) than are *S. virgatus* male bellies, particularly in terms of brightness. A principal component analysis identified two axes describing 94% of the variation in contrast between these two species and their habitats. Using a MANOVA to test for species differences in these two PC axes, we found an overall difference supported by a species difference in PC1 (corresponding to brightness reflectance). *S. siniferus* bellies contrast more with the substrate than do *S. virgatus* bellies. See Results for additional details.

**Figure 5.**
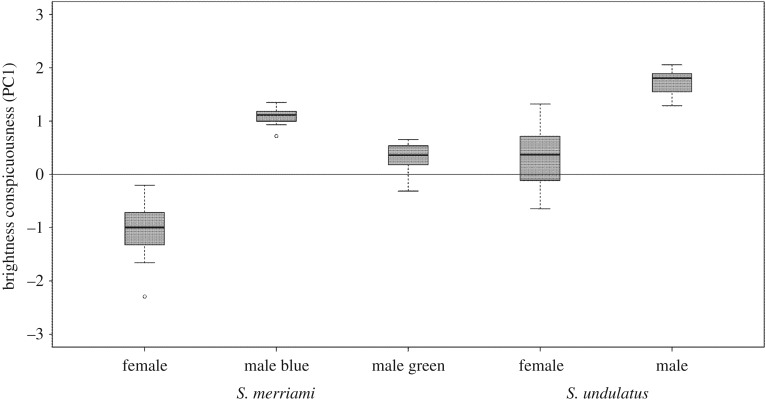
Sex differences are pronounced in both of the two species with colourful male belly patches. A MANOVA used for testing for sex differences in the two PC axes found a significant interaction between clade, colour and sex, with *post hoc* tests confirming significant sex differences in *S. undulatus* and *S. merriami.* See Results for additional details.

This pattern was reflected also in the raw spectral values. A PCA summarizing differences between raw spectral measures of males and females of all four species identified two major axes that together described 94% of the variation: one corresponding to brightness across the spectrum and explaining 66% of the variation, and a second axis emphasizing UV (320–420 nm) reflectance and explaining 34% of the variation. *Sceloporus virgatus* males were very similar to their backgrounds (pale rocks) in terms of brightness, whereas *S. siniferus* males were much brighter than the dark logs typical of their habitat (figures 2 and 4). Males of the two species with colourful bellies were also conspicuous, but tended to be darker than their substrates (figures 2 and 5). Thus, a MANOVA comparing conspicuousness of males from all four species in both PC axes simultaneously found a significant interaction effect between clade and colour (*F*_1,121_ = 273.6; *p* < 0.001), supported by a significant clade (the clade representing the more ancient shift were brighter than those representing the more recent shift: *F*_1,121_ = 458.6; *p* < 0.001) and a significant main effect of colour (species with colourful belly patches tended to be darker than the substrate, whereas species with white bellies were lighter than the substrate: *F*_2,121_ = 53.7; *p* < 0.001).

### 3.3. White bellies from recent shift are sexually monomorphic; those from ancient shift are sexually dimorphic

Although *S. siniferus* males and females look quite similar to a human eye, the males are much more conspicuous from the perspective of a lizard receiver. When filtered through a lizard visual model, *S. siniferus*, exhibited the greatest sex differences in perceived conspicuousness, whereas *S. virgatus* males and females were quite similar in conspicuousness, and the two species with colourful male belly patches were intermediate (figure 3). Thus, an ANOVA comparing JND values for both sexes in all four species yielded a significant interaction effect between clade, colour and sex (*F*_1,188_ = 726.2; *p* < 0.001). Both *S. undulatus* and *S. siniferus* males were more conspicuous than were females (Tukey *post hoc* tests: *p* < 0.001). Surprisingly, both male and female *S. merriami* had relatively conspicuous bellies (figure 3), with blue, green and white patches being roughly equivalent in terms of conspicuousness (figure 3). Neither sex difference in *S. merriami* was statistically significant (Tukey *post hoc* tests: *p* = 0.9 for blue, *p* = 0.1 for green). *Sceloporus virgatus* bellies were the least conspicuous of all four species (figure 3), and also did not exhibit any sex difference in conspicuousness (Tukey *post hoc* tests: *p* = 1.0).

These patterns were not fully reflected in the raw spectral data, where *S. siniferus* males and females differed little in conspicuousness, and there was a clear sex difference in *S. merriami* conspicuousness (figures 4 and 5). A MANOVA comparing a PCA conspicuousness analysis for both sexes in all four species again yielded a significant interaction effect between clade, colour and sex (*F*_1,190_ = 7.3; *p* < 0.001), reflecting pronounced sex differences in the two species with colourful male belly patches, and a lack of sex differences in the two white-bellied species. Both male and female *S. siniferus* were generally brighter than their substrates, with no hint of a sex difference in terms of overall brightness conspicuousness (Tukey *post hoc*: *p* = 1.0). The sex difference in JNDs (above) appears to be driven primarily by differences in the UV portion of the spectrum in which both sexes were darker than the substrate, and males were marginally darker than females (figure 2; Tukey *post hoc* for sex difference in UV conspicuousness: *p* = 0.11). In other words, the *S. siniferus* visual system exaggerates the subtle physical difference between the colours of male and female bellies.

As with the JNDs above, *S. undulatus* males were darker and more conspicuous than were *S. undulatus* females when described by the PC spectral analysis (figure 5), significantly so in terms of brightness (Tukey *post hoc*: *p* < 0.001) and marginally significant in terms of UV reflectance (Tukey *post hoc*: *p* = 0.06). The PC spectral analysis also makes it clear that there is a sex difference in the colour of *S. merriami*. The blue of *S. merriami* males and *S. merriami* females were roughly equal in conspicuousness (as in above JNDs) but in opposite directions (males were darker than the substrate, whereas females were lighter, figure 5), leading to a significant sex difference in terms of brightness conspicuousness (Tukey *post hoc*: *p* < 0.001) and marginally significant sex difference in terms of UV conspicuousness (Tukey *post hoc*: *p* = 0.08). The green of *S. merriami* males was remarkably similar to its background in absolute terms (figure 4) and so much less conspicuous than *S. merriami* females in terms of PC brightness (Tukey *post hoc*: *p* < 0.001), and more conspicuous than *S. merriami* females in terms of UV reflectance (Tukey *post hoc*: *p* < 0.001). Thus, in a raw, physical sense, although both the blue and green of *S. merriami* belly patches are sexually dimorphic and potentially useful as signalling colours, the blue is quite conspicuous against the substrate, whereas the green is remarkably inconspicuous, even in comparison with the female belly. In other words, the new green element is both inconspicuous in an absolute physical sense, and easily visible to a lizard receiver.

## 4. Discussion

Together, our results are consistent with the hypothesis that the blue belly patches of male *Sceloporus* lizards have been lost repeatedly (seven times according to our reconstruction). The bellies of *S. virgatus* males, a species that has lost the blue patch relatively recently, closely resemble the habitat in which they are found, suggesting that natural selection may be responsible for the loss and is capable of having a very fast impact, making these lizards less conspicuous to predators or other unwanted receivers. In contrast, our detailed spectral analyses suggest that sexual selection may have produced new colours over longer periods of time. *Sceloporus siniferus* males, which derive from ancestors that probably lost the sexually dimorphic blue patch many millions of years ago, have evolved a new conspicuous signal that is not present in *S. siniferus* females: a white belly that is darker than the substrate in the UV parts of the spectrum. *Sceloporus merriami* males have added a new green element to their belly patches, a colour that is as sexually dimorphic as the blue colour, but which is also much less conspicuous in comparison with the habitat, in an absolute sense. These results emphasize the importance of a historical approach as well as objective colour measures that take into account also receiver physiology.

Our results confirm that the *Sceloporus* blue belly patches are more conspicuous against the substrate than are plain white bellies in one species comparison reflecting a recent loss of blue (*S. undulatus* versus *S. virgatus*), supporting the hypothesis that male belly patches are signals that have been lost owing to predation pressure. The fast impact of natural selection is further supported by Quinn & Hews's [63] finding that *S. virgatus* males respond appropriately to blue belly patches, despite not having any of their own. In *Anolis* lizards, sexual selection has primarily shaped the evolution of visual signals by making them more conspicuous against different noise and light environments [27,28,64,65]. However, natural selection can also lead to a decrease in conspicuousness, as with *Anolis sagrei* which perform fewer conspicuous motion displays (i.e. push-ups) after recent experience with a predator [66]. In *Sceloporus*, the loss of blue belly patches is also accompanied by an increase in the use of dynamic motion displays [67] and a more dramatic response to chemical cues [68], both giving the white-bellied lizards more direct control over the specific contexts in which they communicate. Further studies of the specific predators experienced by each species are needed to determine exactly how their visual systems may have shaped the evolution of lizard colours, and to refute the alternative possibility that the belly colours were lost owing to forces unrelated to predators.

Our results, however, also emphasize the importance of caution in both assuming that all plain white surfaces are the same, and in applying only a visual model that incorporates receiver sensory system to interpret differences in conspicuousness. For example, although *S. siniferus* males and females have plain white bellies as viewed by the human eye or as measured in an absolute spectral sense, they are remarkably sexually dichromatic from the perspective of a lizard receiver. In this case, our finding adds to the growing body of evidence that the human visual system is insufficient for classifying animal coloration [69–71]. In general, diurnal lizards are similar to birds in having ultraviolet photoreceptors and oil droplets, which allow them to detect and discriminate colours in the ultraviolet (see reviews [39,51]). A few other lizard species exhibit sexually dimorphic UV colours that function in male–female [72] or male–male [73–75] interactions. It is particularly interesting that *S. siniferus* males have reduced rather than enhanced UV reflectance on their bellies. Additional studies are needed to determine if the reduced UV is shaped into a patch or other pattern and whether those patches serve a signalling function. In contrast, using only the visual model, we found that blue, green and white elements of the *S. merriami* belly colours are equally conspicuous to a lizard's eye. We needed a detailed spectral analysis of the raw light measurements to show that although the colours are equally different from the background, they would be quite distinct to a receiver, because the differences are in opposite directions with females being brighter and males darker than the substrate. In both *S. siniferus* and *S. merriami*, belly colours have evolved sex differences that are especially visible to a lizard receiver, while also being much less conspicuous in an absolute sense. Future studies incorporating visual models of snake or other predators may also be important.

Recent comparative studies have examined how trade-offs between natural and sexual selection have helped to shape the evolution of sexual dichromatism [1,74,75]. Our results suggest that the ways in which each type of selection shapes colour signals is more complex than a trade-off. We find evidence that suggests that natural selection has an immediate, lasting impact leading to the loss of a conspecific signal. In contrast, sexual selection may have contributed to the addition of new signals that target receiver sensory systems while remaining inconspicuous in an absolute sense. Additional studies of the belly colours of closely related species may help to confirm the suggestion that sexual selection has a slower, accumulating impact or to eliminate the alternative possibility that the ancestor of *S. siniferus* exhibited unusually high levels of conspicuousness and sexual dimorphism. Similarly, we need additional studies of the pinks, greens, oranges and blacks exhibited by other *Sceloporus* species to confirm that sexual selection is an important force in shaping these novel colours. Over such long periods of time, it is also possible that shifting selective pressures and consequent phenotypic changes have counteracted each other, erasing all evidence of the earliest forces. More comparative studies considering changes in signal colours over long periods of time are needed to confirm the generality of our findings.

Our results are consistent with those from grackles and fairy wrens, which indicate that sexual selection shapes signals slowly whereas natural selection acts quickly in bursts [12,13]. In contrast, natural selection appears to act slowly in shaping strawberry poison dart frog aposematic coloration, with sexual selection acting over shorter periods of time [15]. How and when selection acts on communicative signals clearly depends on the nature of the signal itself, as well as the intended receivers, and future studies should aim to integrate these factors in a comparative framework to better understand how a complex selective regime can shape the evolution of animal signals.

## Data accessibility

Data from this publication have been archived in the Dryad Digital Repository at http://dx.doi.org/10.5061/dryad.d05h5 [76].
